# Supplementary material for: Transcriptome and Functional Comparison of Primary and Immortalized Endothelial Cells of the Human Choroid Plexus at the Blood–Cerebrospinal Fluid Barrier
Source: Int J Mol Sci. 2025 Feb 19;26(4):1779. doi: 10.3390/ijms26041779 (PMC11856769; doi:10.3390/ijms26041779)
Supplement: Supplementary file 1 [file ijms-26-01779-s001.zip › suppl files/Suppl Figures/Suppl_Fig1_Denzer_et_al_2025.pdf]

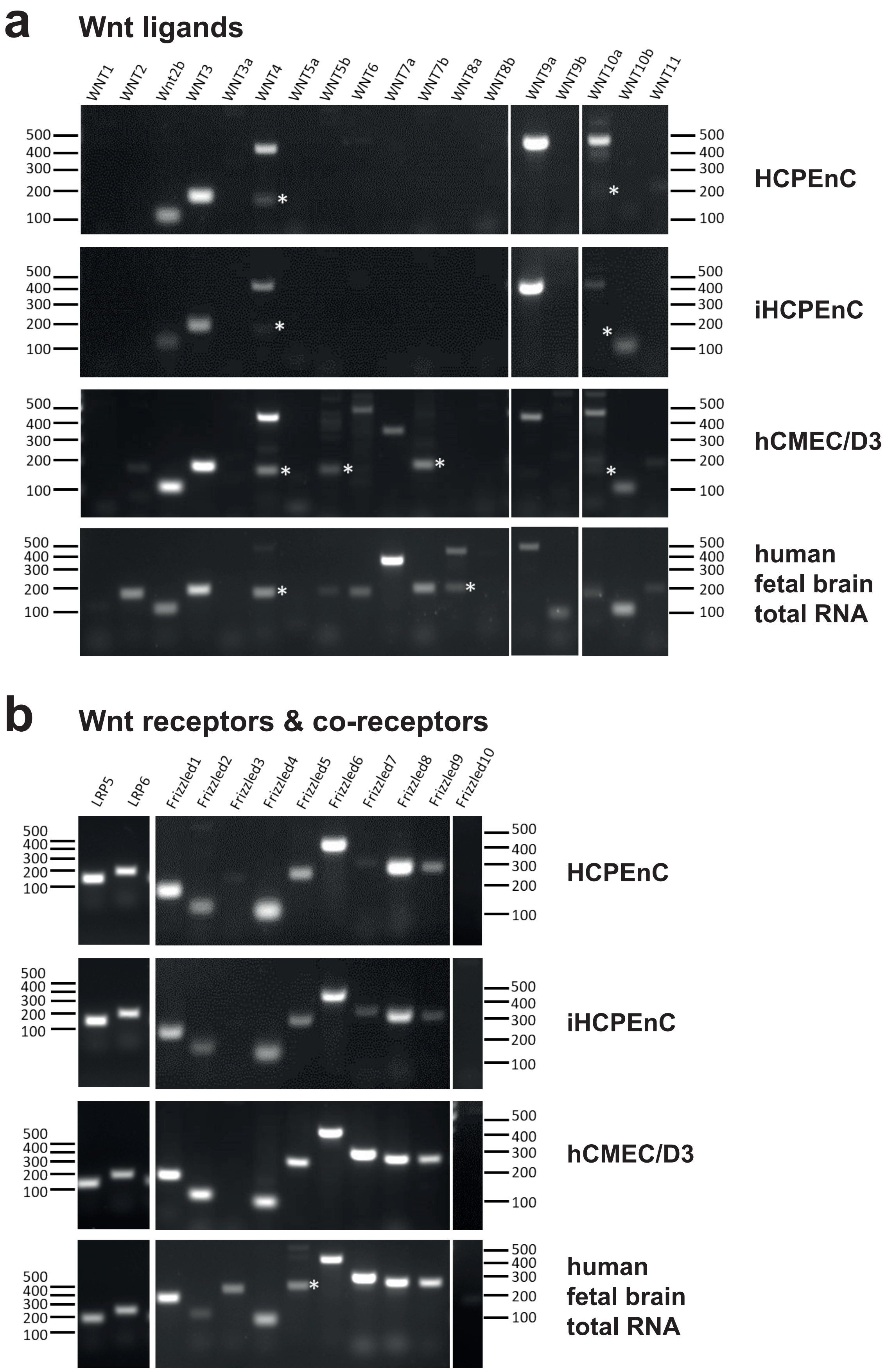

Supplementary Figure 1. Expression of the canonical Wnt signaling pathway in HCPEnC and iHCPEnC; Wnt ligands, Wnt receptors, and co-receptors.
The expression of (a) Wnt ligands, and (b) Wnt receptors and co-receptors in HCPEnC and iHCPEnC was determined by PCR. hCMEC/D3 and human fetal brain total RNA were used as controls. The asterisks indicate the expected positions of amplificates when additional signals were obtained. Data show a representative result of at least three independent experiments (n=3).
