# Supplementary material for: Transcriptome and Functional Comparison of Primary and Immortalized Endothelial Cells of the Human Choroid Plexus at the Blood–Cerebrospinal Fluid Barrier
Source: Int J Mol Sci. 2025 Feb 19;26(4):1779. doi: 10.3390/ijms26041779 (PMC11856769; doi:10.3390/ijms26041779)
Supplement: Supplementary file 1 [file ijms-26-01779-s001.zip › suppl files/Suppl Figures/Suppl_Fig3_Denzer_et_al_2025.pdf]

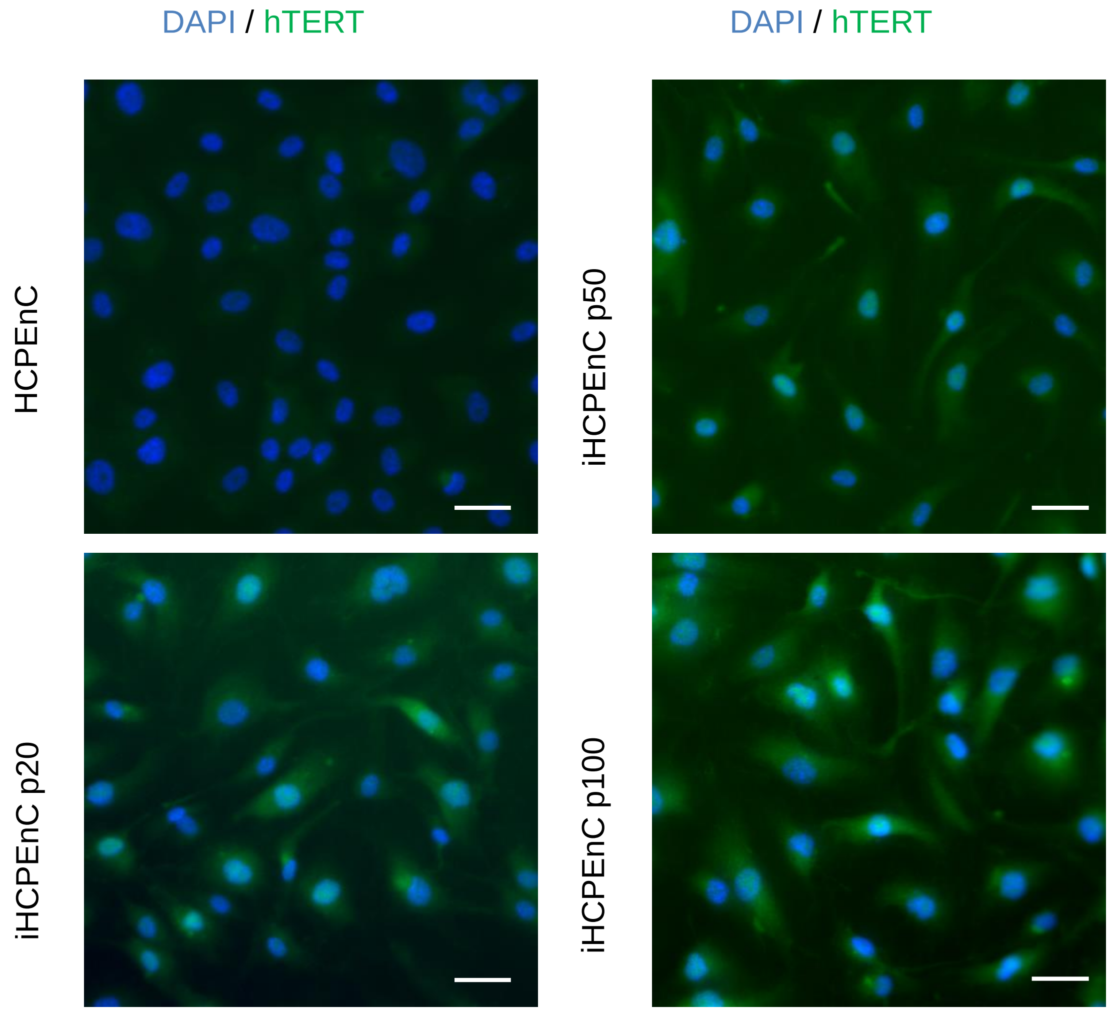

Supplementary Figure 3. Expression of extrinsic hTERT in HCPEnC and iHCPEnC. HCPEnC and iHCPEnC up to passage 100 (p20, p50, p100) were stained with an antibody against hTERT (green). Nuclei were stained with DAPI (blue). Data show a representative result of at least three independent experiments (n=3). Size bar: 40 µm.
