# Supplementary material for: Transcriptome and Functional Comparison of Primary and Immortalized Endothelial Cells of the Human Choroid Plexus at the Blood–Cerebrospinal Fluid Barrier
Source: Int J Mol Sci. 2025 Feb 19;26(4):1779. doi: 10.3390/ijms26041779 (PMC11856769; doi:10.3390/ijms26041779)
Supplement: Supplementary file 1 [file ijms-26-01779-s001.zip › suppl files/Suppl Tables/Denzer_et_al_2025_suppl_Table_S5.pdf]

Supplementary Table S5. Primers used for RT-PCR and qRT-PCR.

Gene symbol	Forward primer	Reverse primer	Size	Reference
**Primers for RT-PCR**				
WNT1	CAAGATCGTCAACCGAGGCT	TCACACGTGCAGGATTCGAT	120	Laksitorini et al. 2019
WNT2	CGTGTGTGCAACCTGACTTC	TGTGTGCACATCCAGAGCTT	168	Laksitorini et al. 2019
WNT2B	GATCCGAGAGTGTCAGCACC	CCTCTCGGCTACTTCTGAGC	107	Laksitorini et al. 2019
WNT3	TGACTCGCATCATAAGGGGC	GTGGTCCAGGATAGTCGTGC	181	Laksitorini et al. 2019
WNT3A	AGCAGGACTCCCACCTAAAC	AGAGGAGACACTAGCTCCAGG	132	Laksitorini et al. 2019
WNT4	TCTTCGCCGTCTTCTCAGCC	GCACCGAGTCCATGACTTCC	162	Laksitorini et al. 2019
WNT5A	TGTTGCTCGGCCCAGAAGTC	GCTTCAATTACAACCTGGGCG	137	Laksitorini et al. 2019
WNT5B	GCGAGAAGACTGGAATCAGGG	TAATGACCACCAGGAGTTGGC	163	Laksitorini et al. 2019
WNT6	CGGGGAGCGTTTAAAGGACA	TTATTGATACTAACCTCACCCACC	159	Laksitorini et al. 2019
WNT7A	AGTACAACGAGGCCGTTCAC	GCACGTGTTGCACTTGACAT	326	Laksitorini et al. 2019
WNT7B	TACGTGAAGCTCGGAGCACT	CGGAACTGGTACTGGCACTC	176	Laksitorini et al. 2019
WNT8A	CTGGTCAGTGAACAATTTCC	GTAGCACTTCTCAGCCTGTT	180	Laksitorini et al. 2019
WNT8B	TATCAGTTTGCCTGGGACCG	CTGTCTCCCGATTGGCACTG	91	Laksitorini et al. 2019
WNT9A	GACGGTCAAGCAAGGATCTG	TGCTCTCGCAGTTCTTCTCA	411	Laksitorini et al. 2019
WNT9B	GTGTCTTGCCATAGCAGGCTT	AATAAGGAGGCCGTGTGTCAG	70	Laksitorini et al. 2019
WNT10A	CTGTTCTTCCTACTGCTGCT	ACACACACCTCCATCTGC	152	Laksitorini et al. 2019
WNT10B	GTCTCCCCACGGTTTAAGCA	TCAGGACCTCCAGTGGTTTG	91	Laksitorini et al. 2019
WNT11	TCTTTGGGGTGGCACTTCTC	TCTGCCGAGTTCACTTGACG	177	Laksitorini et al. 2019

LRP5	ATGGGCGCCAGAACATCAA	AGATGTCGATGCTGAGGTCGTG	117	Laksitorini et al. 2019
LRP6	TTGTTGCTTTATGCAAACAGACG	CGTTTAATGGCTTCTTCGCTGAC	167	Laksitorini et al. 2019
FZD1	GTGAGCCGACCAAGGTGTAT	CAGCCGGACAAGAAGATGAT	184	Laksitorini et al. 2019
FZD2	GCGAAGCCCTCATGAACAAG	TCCGTCCTCGGAGTGGTTCT	116	Laksitorini et al. 2019
FZD3	TGAGTGTTCGAAGCTCTATGG	ATCACGCACATGCAGAAAAG	229	Laksitorini et al. 2019
FZD4	CAGTGAGGCATGGAGGTGTT	AAAGAGCTCAAGGGGCCATC	95	Laksitorini et al. 2019
FZD5	TACCCAGCCTGTCGCTAAAC	AAAACCGTCCAAAGATAAACTGC	247	Laksitorini et al. 2019
FZD6	TGGCCTGAGGAGCTTGAATGTGAC	TATCGCCCAGCAAAAATCCAATGA	421	Laksitorini et al. 2019
FZD7	GTTTGGATGAAAAGATTTCAGGC	GACCACTGCTTGACAAGCACAC	295	Laksitorini et al. 2019
FZD8	ACAGTGTTGATTGCTATTAGCATG	GTGAAATCTGTGTATCTGACTGC	269	Laksitorini et al. 2019
FZD9	CCCTAGAGACAGCTGACTAGCAG	CGGGGGTTTATTCCAGTCACAGC	264	Laksitorini et al. 2019
FZD10	ACACGTCCAACGCCAGCATG	ACACGTCCAACGCCAGCATG	170	Laksitorini et al. 2019
WNT16	TCAGGGAGACCCTCTTCACAG	AGCAGGTACGGTTTCCTCTTG	151	Laksitorini et al. 2019
sFRP1	TGGCCCGAGATGCTTAAGTG	CCTCAGTGCAAACTCGCTGG	180	Laksitorini et al. 2019
sFRP2	CTCGCTGCTGCTGCTCTTC	GGCTTCACATACCTTTGGAG	505	Laksitorini et al. 2019
sFRP3	ATGGTCTGCGGCAGCCCGG	CTGTCGTACACTGGCAGCTC	431	Laksitorini et al. 2019
sFRP4	GTTCCTCTCCATCCTAGTGG	GCTGAGATACGTTGCCAAAG	574	Laksitorini et al. 2019
sFRP5	CTACTGGAGGGTGTTTTCAC	CTTTCCCTTACCCTCTCCT	201	Laksitorini et al. 2019
WIF1	CACCTGGATTCTATGGAGTG	ACAGAGGTCTCCCTGGTAAC	218	Laksitorini et al. 2019
DKK1	CAGGATTGTGTTGTGCTAGA	TGACAAGTGTGAAGCCTAGA	202	Laksitorini et al. 2019
DKK2	CTCAACTCCATCAAGTCCTC	TACCTCCCAACTTCACACTC	164	Laksitorini et al. 2019

DKK3	GAGGTTGAGGAACTGATGG	CCAGTCTGGTTGTTGGTTAT	215	Laksitorini et al. 2019
DKK4	GTCCTGGACTTCAACAACAT	GTTGCATCTTCCATCGTAGT	233	Laksitorini et al. 2019
PLVAP	CTGCGATGCCTTGCTCTTCAT	AGTCCCTCCACAGGTTACGA	272	Laksitorini et al. 2019
OCLN	AAGCAAGTGAAGGGATCTGC	GGGGTTATGGTCCAAAGTCA	204	Laksitorini et al. 2019
GLUT1	AATACACCACCTCACTCCTG	GAGGTACGTGTAAGGGACTG	188	Laksitorini et al. 2019
BCRP	CAGTCTTCAAGGAGATCAGC	CCAGTACGACTGTGACAATG	132	Laksitorini et al. 2019
P-GP	ATATCAGCAGCCCACATCAT	GAAGCACTGGGATGTCCGGT	154	Laksitorini et al. 2019
CDH5	GTTCGGCTGACAGGTCCACA	CGATGTGGCGAGGAGCATCA	147	Laksitorini et al. 2019
ZO-1	ATCTCGGAAAAGTGCCAGGA	TTTCAGCGCACCATACCAAC	124	Laksitorini et al. 2019
CLDN5	AGGCGTGCTCTACCTGTTTTG	AACTCGCGGACGACAATGTT	78	Laksitorini et al. 2019
CLDN3	GCCACCAAGGTCGTCTACTC	CGTAGTCCTTGCGGTCGTAG	82	Laksitorini et al. 2019
CLDN1	TTTACTCCTATGCCGGCGAC	GAGGATGCCAACCACCATCA	173	Laksitorini et al. 2019
CCND1	GTCCCACTCCTACGATACGC	CAGGGCCGTTGGGTAGAAAA	129	Laksitorini et al. 2019
APCDD1	AAGGAGTCACAGTGCCATCA	TTGTGATGAACTCTGGGCCT	136	Laksitorini et al. 2019
AXIN-2	GACAGGAATCATTCGGCCAC	CCTTCAGCATCCTCCGGTAT	181	Laksitorini et al. 2019
CTNNB	TCTGAGGACAAGCCACAAGATTACA	TGGGCACCAATATCAAGTCCAA	122	Laksitorini et al. 2019
**Primers for qRT-PCR**				
VWF	CGGCTTGCACCATTCAGCTA	TGCAGAAGTGAGTATCACAGCCATC	90	Xu et al. 2017
PLAT	GGTCTGGAGAAGTCTGTAGAG	CCTAGACTGGATTCGTGACAA	134	Yan et al. 2020
THBS1	AGTGGAAGAGCATCACGCTG	CACCACGTTGTTGTCAAGGG	287	Bray et al. 2019
SERPINE1	GCAAGGCACCTCTGAGAACT	GGGTGAGAAAACCACGTTGC	202	Zhang et al. 2020

EDN1	CAGAAGAAGTTCAGAGGAACACC	GGAAGCCAGTGAAGATGGTT	334	Lee et al. 2018
RPL13a	GGATGAACACCAACCCTTCC	AACACCTTGAGACGGTCCAG	133	Kang et al. 2019
SDHA	CATCCACTACATGACGGAGCA	ATCTTGCCATCTTCAGTTCTGCTA	90	Modesto et al. 2013

References

Bray ER, Yungher BJ, Levay K, Ribeiro M, Dvoryanchikov G, Ayupe AC, Thakor K, Marks V, Randolph M, Danzi MC, Schmidt TM, Chaudhari N, Lemmon VP, Hattar S, Park KK. Thrombospondin-1 Mediates Axon Regeneration in Retinal Ganglion Cells. Neuron. 2019 Aug 21;103(4):642-657.e7. doi: 10.1016/j.neuron.2019.05.044.

Kang IN, Lee CY, Tan SC. Selection of best reference genes for qRT-PCR analysis of human neural stem cells preconditioned with hypoxia or baicalein-enriched fraction extracted from Oroxylum indicum medicinal plant. Heliyon. 2019 Aug 1;5(7):e02156. doi: 10.1016/j.heliyon.2019.e02156.

Laksitorini MD, Yathindranath V, Xiong W, Hombach-Klonisch S, Miller DW. Modulation of Wnt/β-catenin signaling promotes blood-brain barrier phenotype in cultured brain endothelial cells. Sci Rep. 2019 Dec 23;9(1):19718. doi: 10.1038/s41598-019-56075-w

Lee H, Kim M, Park YH, Park JB. Dexamethasone downregulates SIRT1 and IL6 and upregulates EDN1 genes in stem cells derived from gingivae via the AGE/RAGE pathway. Biotechnol Lett. 2018 Mar;40(3):509-519. doi: 10.1007/s10529-017-2493-0.

Modesto P, Peletto S, Pisoni G, Cremonesi P, Castiglioni B, Colussi S, Caramelli M, Bronzo V, Moroni P, Acutis PL. Evaluation of internal reference genes for quantitative expression analysis by real-time reverse transcription-PCR in somatic cells from goat milk. J Dairy Sci. 2013;96(12):7932-44. doi: 10.3168/jds.2012-6383.

Xu Y, Pan S, Liu J, Dong F, Cheng Z, Zhang J, Qi R, Zang Q, Zhang C, Wang X, Zhang J, Wang F, Allen TD, Liu J. GATA3-induced vWF upregulation in the lung adenocarcinoma vasculature. Oncotarget. 2017 Nov 30;8(66):110517-110529. doi: 10.18632/oncotarget.22806.

Yan M, Wang W, Zhou J, Chang M, Peng W, Zhang G, Li J, Li H, Bai C. Knockdown of PLAT enhances the anticancer effect of gefitinib in non-small cell lung cancer. J Thorac Dis. 2020 Mar;12(3):712-723. doi: 10.21037/jtd.2019.12.106.

Zhang Q, Lei L, Jing D. Knockdown of SERPINE1 reverses resistance of triple-negative breast cancer to paclitaxel via suppression of VEGFA. Oncol Rep. 2020 Nov;44(5):1875-1884. doi: 10.3892/or.2020.7770.
